# Synergistic Effect of *Azotobacter nigricans* and Nitrogen Phosphorus Potassium Fertilizer on Agronomic and Yieldtraits of Maize (*Zea mays* L.)

**DOI:** 10.3389/fpls.2022.952212

**Published:** 2022-08-03

**Authors:** Alka Sagar, R. Z. Sayyed, Pramod W. Ramteke, Wusirika Ramakrishna, Peter Poczai, Sami Al Obaid, Mohammad Javed Ansari

**Affiliations:** ^1^Department of Biotechnology, Meerut Institute of Engineering and Technology, Meerut, India; ^2^Department of Microbiology, PSGVP Mandal’s S. I. Patil Arts, G. B. Patel Science and S. T. K. V Sangh Commerce College, Shahada, India; ^3^Department of Biotechnology, Dr. Ambedkar College, Nagpur, India; ^4^Department of Biochemistry, Central University of Punjab, Bathinda, India; ^5^Finnish Museum of Natural History, University of Helsinki, Helsinki, Finland; ^6^Department of Botany and Microbiology, College of Science, King Saud University, Riyadh, Saudi Arabia; ^7^Department of Botany, Hindu College, Moradabad (Mahatma Jyotiba Phule Rohilkhand University Bareilly), Moradabad, India

**Keywords:** *Azotobacter nigricans*, ACC deaminase, catalase, glutathione reductase, superoxide dismutase (SOD), maize, NPK

## Abstract

Plant growth-promoting bacteria (PGPB) *Azotobacter* spp. is the most promising bacteria among all microorganisms. It is an aerobic, free-living, and N2-fixing bacterium that commonly lives in soil, water, and sediments. It can be used as a biofertilizer for plant growth and nutrient utilization efficiency. Maize is the highly consumed cereal food crop of the cosmopolitan population, and the sustainable maize productivity achieved by applying bacteria in combination with nitrogen phosphorus potassium (NPK) is promising. In the present study, a bacterial isolate (PR19). *Azotobacter nigricans*, obtained from the soil of an organic farm was evaluated for its plant growth promoting potential alone and in combination with an inorganic fertilizer (NPK) included. The bacterial cultue (PR19) was screened for its morphological, biochemical, and plant growth-promoting characteristics, sequenced by the 16S rDNA method, and submitted to NCBI for the confirmation of strain identification. Further, the inoculation effect of the bacterial culture (PR19) in combination with NPK on growth and yield parameters of maize under pot were analyzed. Based on phenotypic and molecular characteristics, PR19 was identified as *Azotobacter nigricans* it was submitted to NCBI genbank under the accession No. KP966496. The bacterial isolate possessed multiple plant growth-promoting (MPGP) traits such as the production of ammonia, siderophore, indole-3-acetic acid (IAA), and ACC Deaminase (ACCD). It showed phosphate solubilization activity and tolerance to 20% salt, wide range of pH 5–9, higher levels of trace elements and heavy metals, and resistance to multiple antibiotics. PR19 expressed significantly increased (*p* < 0.001) antioxidant enzyme activities (SOD, CAT, and GSH) under the abiotic stress of salinity and pH. *In vitro* condition, inoculation of maize with the PR19 showed a significant increase in seed germination and enhancement in elongation of root and shoot compared to untreated control. The combined application of the PR19 and NPK treatments showed similar significant results in all growth and yield parameters of maize variety SHIATS-M S2. This study is the first report on the beneficial effects of organic farm isolated PR19-NPK treatment combinations on sustainable maize productivity.

## Introduction

While macronutrients such as nitrogen (N), phosphorus (P), potassium (K), and sulfur (S) supplied by mineral fertilizers are important for crop production, agriculturally beneficial microorganisms can also contribute directly or indirectly for crop improvement. Use of environmentally friendly, sustainable, innovative nutrient management approaches that emphasize on restoration and maintenance of soil quality, less resilience on chemical fertilizers to combat adverse effects of low soil fertility, soil acidification, and nitrate leaching into groundwater both in the short and long term are is the need of the hour. Thus, effective biological techniques such as plant growth-promoting bacteria (PGPB) are used to augment crop productivity (Dinesh et al., 2013). PGPB have been seen as one of the best bio-stimulants and bio-inoculants to improve crop productivity (Kalam et al., 2020; Basu et al., 2021; Hamid et al., 2021; Kannepalli et al., 2021; Khan et al., 2021; Manasa et al., 2021). PGPB is partially used as part of some bio-fertilizers to replace chemical fertilizers; they make up only a small fraction of fertilizer used in global agriculture (Yakhin et al., 2017). Although PGPB characterization is ongoing, it is still necessary to identify new strains adapted to native soils and climates (Backer et al., 2018).

There are several PGPB accessible to boost soil fertility; one is *Azotobacter*. It is a free-living Nitrogen–fixer diazotroph (an average of 20 kg N/ha/per year) and ultimately boosts up biological nitrogen fixation that is extremely diverse and globally prevalent in soils and represents the dominant natural source of N in ecosystems lacking SNF (Jnawali et al., 2015; Aasfar et al., 2021). Additionally, the availability of *Azotobacter* sp. in the soil could enhance the availability of Nitrogen (Din et al., 2019), and P (Velmourougane et al., 2019). Among the phosphate-solubilizing bacteria (PSB), *Bacillus* and *Pseudomonas* are the most common. Some *Azotobacter* sp. are, also known for their P solubilizing capacity (Ramakrishna et al., 2020; Yadav et al., 2020a; Aasfar et al., 2021).

In addition, it stimulates rhizospheric microbes, protects the plants from pathogens, and improves nutrient uptake (Hamid et al., 2021; Sarkar et al., 2021), i.e., soil carbon and sulphur contents are increased in response to inoculation with *Azotobacter* sp. by accelerating the mineralization of soil organic residues, which subsequently reduced heavy metals absorption by roots (Kizilkaya, 2009). The nitrogen supply to the plant will influence the amount of protein, amino acids, protoplasm, and chlorophyll formed. Therefore, an adequate supply of nitrogen is necessary to achieve high yield potential in the crop. *Azotobacter* sp. probably influences the development of plants by producing growth–regulating substances. It helps in the synthesis of growth-regulating substances like auxins, cytokinin, and Gibberellic Acid (GA), and these growth materials are the primary substance controlling the enhanced growth. *Azotobacter sp.* produced Indol–3–Acetic Acid (IAA) when tryptophan was added to the medium (Khan et al., 2020; Khairnar et al., 2022). Cytokinins are related to nucleic acids with precursor substances that act to stimulate cell division in vegetative growth areas. *Azotobacter* sp. are capable of producing siderophore; they bind to the available form of iron Fe + 3 in the rhizosphere, thus making it unavailable to the phytopathogens and protecting the plant health in addition to Hydrogen Cyanide (HCN) production (higher in *Azotobacter* −77.00%; Nithyapriya et al., 2021); exo-polysaccharide production (Sheikh et al., 2022).

*Azotobacter* sp. secretes an antibiotic with a structure similar to anisomycin, which is a documented fungicidal antibiotic. *Azotobacter* sp., in sufficient numbers, will out-compete with pathogens for food. Some of the pathogens that have been controlled by *Azotobacter* sp. in the soil and on the leaf (Kalam et al., 2020; Sukmawati et al., 2021) and ease abiotic stress in plants (Sagar et al., 2020a, 2022a,b; Kusale et al., 2021a,b). *Azotobacter* sp. possess some unique features, such as cyst formation conferring resistance to environmental stresses to develop specific *Azotobacter* sp. cyst-based formulations (Aasfar et al., 2021).

Studies have shown that PGPB positively affects grains, fruits, vegetables, and spices. PGPR has also been found to improve plant uptake of nutrients and thus increase the use efficiency of applied fertilizers and manure for lower application rates of fertilizers. With this preview, the present study aimed to evaluate the plant-growth promotion potential of the salt-tolerant *Azotobacter nigricans* (KP966496) PR19 isolated from the organic farm of Sam Higginbottom University of Agriculture, Technology and Sciences (SHUATS), Allahabad, India, and its synergistic effect with nitrogen phosphorus potassium (NPK) to enhance agronomic traits in maize under pot conditions.

## Materials and Methods

### Collection of Soil Samples and Isolation of Bacteria

A 10 g of soil sample was collected from different sites of SHUATS Model Organic Farm (SMOF) Allahabad, India (25° 24′ 42″ N, 81° 50′ 56″ E, and 98 m altitude above the mean sea level (Sagar et al., 2017). The sample was placed in sterile plastic bags, kept at 40°C in the laboratory, and analyzed within 4 h of collection. The soil sample was serially diluted in sterile phosphate-buffered saline (Hi-media, pH 7.2) and inoculated on Ashby’s Agar for *Azotobacter*ia sp. (Norris and Chapman, 1968).

### Morphological and Biochemical Identification of Bacterial Culture

The bacterial culture was identified by its morphological and biochemical characteristics, including Gram staining, oxidase test, indole test, methyl red test, Voges–Proskauer test, citrate utilization, glucose fermentation, acid from lactose, H_2_S production, sugar fermentation, acetoin production, catalase activity and Gelatin liquefaction (Aneja, 2001).

### Molecular Characterization of Isolate

DNA extraction, partial 16S rRNA gene amplification of bacterial culture, PCR product purification, and following sequencing analysis was performed according to Rademaker et al. (1998). Universal primers, PF (5′-TGGCTCAGATTGAACGCTGGCGG-3′) and PR (5′-GGCTCAGATTGAACGCTGGCGG-3′) were used to amplify the 16S rRNA gene (Edwards et al., 1989). The amplified and purified PCR fragments were sequenced in ABI3100 Genetic Analyzer with the same primers. The sequences were compared with 16S rRNA gene sequences available in the NCBI GenBank database using the BLASTn programme. The species level identified as maximum homology (≥97%) to a type strain sequence in the GenBank.

### Plant Growth-Promoting Traits of Isolated Bacteria

#### Production of Indole-3-AceticAcid

The production of IAA was detected as described by Brick et al. (1991). The bacterial culture was grown in peptone water for 72 h at 37°C. Fully grown cultures were centrifuged at 3000 rpm for 30 min. The supernatant (2 ml) was mixed with two drops of orthophosphoric acid and 4 ml of Salkowski reagent. The absorbance was read at 530 nm. IAA quantification was performed by preparing a calibration curve using IAA as the standard.

#### Phosphate Solubilization

The bacterial culture was grown in Pikovskaya’s agar medium and incubated at 28°C for 4–5 days and observed for the presence of a transparent halo around the organism indicated PS activity (Nautiyal, 1999).

#### Siderophore production

Log phase bacterial culture was grown on ChromeazurolSulphante (CAS) agar plate at 30°C for 48 h. Following the incubation, inoculated plates were observed for the presence of an orange halo around the colonies on blue agar (Schwyn and Neilands, 1987; Patel et al., 2018).

#### Ammonia (NH_3_) Production

Ammonia (NH_3_) production was analyzed by inoculating the freshly grown bacterial culture in 10 ml of peptone water and incubating for 48–72 h followed by the addition of 0.5 ml of Nessler’s reagent. Development of brown to yellow color was positive for ammonia production (Cappuccino and Sherman, 1992).

### Biological Control Attributes of Bacterial Culture

#### Hydrogen Cyanide (HCN) Production

HCN production was detected using the method of Bakker and Schippers (1987). King’s medium was amended with 4.4 g glycine l-1, and the bacterial culture was streaked on the agar plate. A Whatman filter paper no. 1 soaked in 2% sodium carbonate and 0.5% picric acid solution was placed at the top of the plate. Plates were sealed with parafilm and incubated at 30°C for 4 days. The development of yellow to red color on the filter paper indicated HCN production.

#### Chitinase Production

Chitinase production was assessed by growing the bacterial culture on M9 chitin agar medium amended with 1% (w/v) colloidal chitin and incubated at 30°C for 24–72. h. Following the incubation, plates were observed for zone of clearance around the growth of isolate (Das et al., 2010).

### Measurement of ACC Deaminase Activity, and Salinity and pH Tolerance

ACC Deaminase activity was performed as described by Safronova et al. (2006). The bacterial culture was grown in the test tube containing 100 ml of liquid medium containing KH2PO4 (2 g), K_2_HPO_4_ (0.5 g), MgSO4 (0.2 g), Glucose (0.2 g). The medium was supplemented with 0.3 g ACC or 0.19 g (NH_4_)_2_SO_4_ as nitrogen source and incubated at 30°C for 24–72 h and observed for the appearance of bacterial growth as an indication of ACC deaminase activity.

The stress tolerance to salinity and pH were studied by separately inoculating the isolate in nutrient broth with 10% salt and in different pH ranges (5–9). Following the incubation at 30°C for 48–72 h each inoculated medium was observed for the luxurious growth of isolate (Damodaran et al., 2013).

#### Tolerance to Heavy Metals and Trace Elements

The agar diffusion method tested the bacterial culture for their tolerance to heavy metals (Cervantes et al., 1986). Freshly prepared agar plates were amended with various soluble heavy metals (Cr, Pb, As, Ag, Au, Hg) and trace elements (Al, Zn, Mo, Mn, Cu, Ni) at various concentrations ranging from 0.6 to 3200 μg/ml and inoculated with the bacterial culture. The incubated plates were kept at 30°C for 48 h, and the effect of heavy metals and trace elements on the growth of the bacterial culture was determined.

#### Susceptibility to Antibiotics

The bacterial culture was tested for its resistance to different antibiotics (viz., Cephalexin, Ampicillin, Cephataxime, Nalidixic, Neomycin, Streptomycin, Vancomycin, Kanamycin, Rifampicin, chloramphenicol, Trimethoprim, Tetracycline, and Gentamycin) by agar diffusion method (Bauer et al., 1966). The bacterial culture was inoculated in freshly prepared agar plates, amended with specific antibiotics, incubated the plates at 37°C for 48 h, and determined the antibiotic resistance by observing the growth of the bacterial culture (Schaad, 1992).

#### Screening for Antioxidant Properties

The antioxidant property of bacterial culture was characterized by measuring the activity of antioxidant enzymes and compounds. Superoxide dismutase (SOD), catalase (CAT), and reduced glutathione oxidase (GSH) are the antioxidant enzymes that protect the plants from oxidative stress damages (Fazeli-Nasab and Sayyed, 2019). The screening and production of SOD, CAT, and GSH in bacterial culture was performed under 20% salt and different pH 5–9 according to the method of Beers and Sizer (1952), Marklund and Marklund (1974), and Salbitani et al. (2017), respectively. One unit of SOD was defined as the amount of the enzyme needed to inhibit 50% of the autoxidation of pyrogallol, one unit of catalase was defined as alM of H_2_O_2_ decomposed per min and one unit of GSH was defied as IM of GSH reduced per min.

### Plant Growth Promotion Studies

#### Maize Varieties

Maize varieties were collected from the Department of Genetics and Plant Breeding, SHUATS, Allahabad. They are listed in the Table 1.

**TABLE 1:** A list of maize varieties used in the present study.

Verities	Remarks
Kanchan	GBPUAT, Pantnager,1982, Kharif
Azad Uttam	C.S. Azad university, Kanpur, 1991, Kharif
Saradmani	C.S. Azad university, Kanpur, 2008, Rabi
Navjyot	PAU, Ludhiana, 1983, Kharif
SHUATS MS-2	State varietal release subcommittee, U.P.

Seed treatment was performed with log phase culture (30 h) of PR19 (10^4^cfu/ml). Treated seeds (10 seeds of each plant) were placed on germination papers in Petri plates at 25°C for 30 days and daily irrigated with water (Nandakumar et al., 2001). Non-bacterized seeds served as control. All the treatments were performed in three replicates, and the average of triplicates was considered. After 15 days of sowing (DOS), the growth parameters were studied under the *in vitro* condition. The percent seed germination was calculated as follows: (number of germinated seeds/number of total seeds) × 100) and root growth (length), shoot growth (length), The germination index (GI) was calculated as described in Association of Official Seed Analysts [AOSA] (1983).


$$\text{GI}=\frac{\text{No}.\mathrm{of}\,\mathrm{germinated}\,\mathrm{seed}}{\mathrm{days}\,\mathrm{of}\,\mathrm{the}\,\mathrm{first}\,\mathrm{count}}+\frac{\text{no}.\mathrm{of}\,\mathrm{germinated}\,\mathrm{seed}}{\mathrm{days}\,\mathrm{of}\,\mathrm{the}\,\mathrm{final}\,\mathrm{count}}\times 100$$

The vigor index (VI) was calculated as follows (Abdul-Baki and Anderson, 1973).


VI=%germination×seedlinglength

Vigor Index by multiplying percent germination by seedling length (shoot length + root length).

### Pot/Polybags Experiment With Bacterial Culture PR19

Based on an *in vitro* study (Especially root and shoot elongation), the SHIATS MS-2 variety of maize was selected for the pot experiment. A pot experiment was conducted at SHUATS, Allahabad. Seeds of the maize variety, SHIATS MS-2, were kept in the PR19 described above the paragraph and sown in pots/polybags containing soil. The soil was placed in polybags (each contained 25–30 kg of soil). These pots were fixed in an open field during the maize growing season (June–September). The experiments were designed in a completely randomized block design (CRBD) with four treatments and three replications. The treatments were defined as follows:

T0 = Control (no-fertilizerand no-bacteria inoculants),
T1 = Std. NPK (120 : 60 : 40 kg/ha) (25%) (no-bacteria inoculants only Fertilizer),
T2 = PR19 (only Bacterial Culture),
T3 = PR19 (Bacterial Culture) and NPKs (120 : 60 : 40 kg/ha).

Plant height (cm) was measured 90 days after sowing (DAS). The average height was based on five plants in each treatment. The flag leaf width was the distance from the middle part to one margin. The flag leaf length was the distance from the collar junction of the blade and leaf sheath to the top of the leaf blade. Grain yield per plant was calculated based on the total number of grains produced by each plant at harvest time. The grain weight was taken using 1000 grains at a time. The fresh weight of a plant was recorded after harvesting. The dry weight was based on the average weight of three plants in each set after 24 h incubation at 60°C. The harvest index (HI) was determined by seed yield per plant/total dry weight per plant × 100.

#### Total Chlorophyll Estimation

Total chlorophyll was estimated as per Arnon (1949). Mature leaves of maize were ground in 80% acetone, centrifuged at 10,000 rpm for 5 min, and acetone was added to the supernatant in a new tube. Absorbance was measured at 645 and 663 nm. The total chlorophyll (μg/ml) was determined as follows: 20.2 (A_645_) + 8.02 (A_663_).

#### Relative Water Content

The relative water content (RWC) was determined as per Barrs and Weatherley (1962). Leaf discs of maize were weighed to record fresh weight. The leaf discs were left on a Petriplate with water for 4 h. The leaf discs were weighed after gentle blotting to record turgid weight. The dry weight was determined after drying in a hot air oven at 72°C for 48 h. RWC calculation was performed as follows


$$\text{RWC}=\frac{\mathrm{fresh}\,\mathrm{weight}-\mathrm{dry}\,\mathrm{weight}}{\mathrm{turgid}\,\mathrm{weight}-\mathrm{dry}\,\mathrm{weight}}\times 100$$

#### Protein Estimation

Protein content in the samples was determined as per the method of Lowry et al. (1951). A standard curve was constructed using bovine serum albumin (BSA) protein. The absorbance was recorded at 660 nm.

#### Carbohydrate Estimation

Mature maize leaves were dried, added to water, and boiled for 1 h. To 1 ml of extract, 3 ml of 3% anthrone reagent (Sigma-Aldrich) was added, followed by incubation at 1000°C for 30 min. Absorbance was recorded at 630 nm with glucose as a standard. The carbohydrate content of the samples was estimated based on the standard curve. The total carbohydrate content was estimated as per Hodge and Hofreiter (1962) and Aneja (2001).

### Statistical Analysis

The experiment was conducted in Completely Randomized Design (CRD). Data given in this report is the average of three replicates that was statistically analyzed using a student t-test. P values of ≤ 0.05 were taken as statistically significant values (Parker, 1979).

## Results

### Identification and Characterization of the Isolate

A total of one hundred and thirty (130) bacterial cultures were isolated from the collected soil samples from SMOF Allahabad (Sagar et al., 2017). Only one (01) bacterial culture (PR19) showed higher tolerance to the salt (20%). The selected bacterial culture was preliminarily characterized based on its morphological, biochemical, MPGP traits, and molecular characterization (Table 2). The bacterial cultureIsolate was Gram-negative rod, motile, and non-acid fast bacteria. The bacterial culture showed a positive reaction to all biochemical parameters, while negative results for Citrate utilization and gelatin liquefaction tests (Table 2).

**TABLE 2:** Morphological and biochemical characterization of *Azotobacter nigricans* (KP966496; PR19).

Characteristics	Characteristics	Positive/Negative
Morphological	Isolation from	Organic farm
	Organism	*Azotobacter nigricans*
	NCBI Accession no	KP966496
	Temperature	37°C
	Gram staining	−
	Shape	Rods
	Motility	+
	Capsule	−
	Spore formation	−
	Flagella	−
	Acid fast staining	+
Biochemical	Oxidase	+
	Indole production	+
	Methyl Red	+
	Voges-proskauer	+
	Citrate utilization	−
	Glucose fermentation	+
	Acid from lactose	+
	Trehalose	+
	Maltose	+
	Cellobiose	+
	H_2_S production	+
	Acetoin production	+
	Catalase	+
	Gelatin liquefaction	−

*+, presence; −, absence.*

**FIGURE 1:**
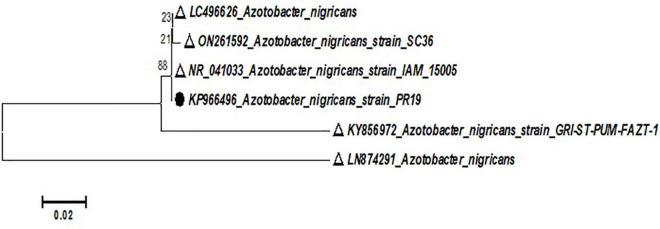
Phylogenetic analysis of PR19 based on 16s rRNA gene sequence drawn using the Neighbor-joining tree (Kimura two-parameter distance) method (MEGA 5.0 software) with evolutionary distances.

Bacterial culture PR19 was identified as the most effective PGPB at the species level. The 16S rRNA sequence was deposited in Gen Bank and sequence was compared after the BLAST on NCBI and phylogenetic tress constructed using MEGA 6.0 software. Similarity values (≥97%) suggested that the bacterial isolate PR19 belongs to the genus *A. nigricans* (Figure 1). The unrooted phylogenetic tree showed the genotypic relationship of the bacterial isolate, where all the bacterial isolates were clustered into two major clades. Thus the bacterial isolate was identified as *Azotobacter nigricans*. The bacterial isolate was submitted to NCBI with the accession number KP966496.

#### Plant Growth Promoting Traits

The selected bacterial culture (PR19)was was screened for multiple plant growth-promoting (PGP) features traits. A clear zone around the colony on the tricalcium phosphate plate indicated the P solubilization ability of the PR19. The PR19 produced siderophore ACC deaminase, and IAA (5.5 μg/ml; Table 3).

**TABLE 3:** PGP traits of *Azotobacter nigricans* (KP966496; PR19).

PGP traits	Positive/Negative
Production of Ammonia (NH_3)_	+
Hydrogen Cyanide (HCN)	−
Siderophore Production (SD)	+
Indole Acetic Acid [IAA(μg/ml)]	5.5
1-aminocyclopropane-1-carboxylate deaminase (ACCD)	+
Phosphorus Solubilization Activity (PS)	+
Chitinase	−
Tolerance to salt%	20
Tolerance to pH	5–9

*+, presence; −, absence.*

**FIGURE 2:**
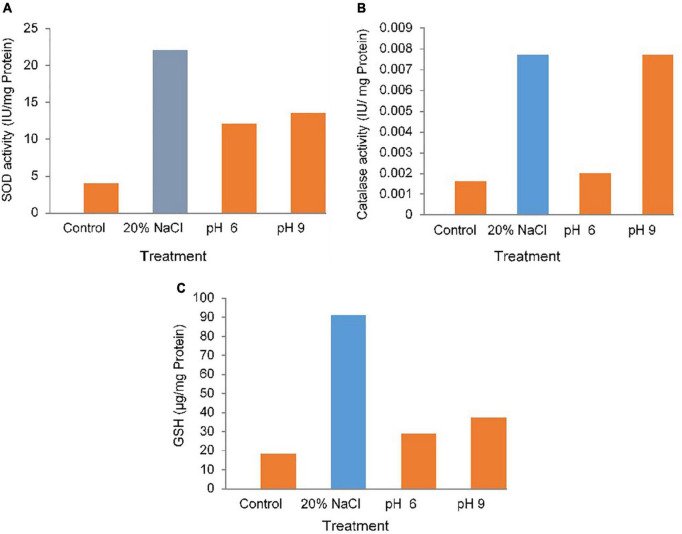
Antioxidant activity **(A)** superoxide dismutase (SOD), **(B)** catalase (CAT), **(C)** glutathione (GSH) of PR19 at various pH (5–9) and high salt (20%) concentrations.

The PR19 grew well upto 20% salt concentration; over the wide pH range (5–9). PR19 showed significant tolerance properties to trace and heavy metal stress (Table 4). Among the trace elements, the highest threshold level (3200 μg/mL) was observed with Aluminum followed by Zine while minimum (2.5 μg/mL) tolerance was observed with mercury (Figure 2).

**TABLE 4:** Tolerance to trace and heavy metals of *Azotobacter nigricans* (KP966496; PR19).

Elements	Heavy metals	Tolerance to μg/ml
Trace elements	Aluminum	3200
	Zinc	1600
	Molybdenum	800
	Manganese	800
	Copper	100
	Nickel	200
Heavy metals	Chromium	200
	Lead	400
	Silver	100
	Arsenic	50
	Gold	25
	Mercury	2.5

It also showed resistance toward Cephalexin (Cp), Ampicillin (A), Streptomycin (S), Vancomycin (V), Kanamycin (K), chloramphenicol (C), and Tetracycline (T), Gentamycin (G), and sensitive against Neomycin (Ne) and Rifampicin (Rf) (Table 5).

**TABLE 5:** Antibiotic susceptibility of *Azotobacter nigricans* (KP966496; PR19).

Antibiotics	Resistance/Sensitive
Cephalexin (Cp)	R
Ampicillin (A)	R
Cephataxime (Cf)	R
Nalidixic (NA)	R
Neomycin (Ne)	S
Streptomycin (S)	R
Vancomycin (V)	R
Kanamycin (K)	R
Rifampicin (Rf)	S
Chloramphenicol (C)	R
Trimethoprim (TM)	R
Tetracycline (T)	R
Gentamycin (G)	R

### Antioxidant Enzyme Activity of PR19 Under Abiotic Stress

The enhancement in the activity/levels of antioxidant enzymes/compounds, SOD, CAT, and GSH in PR19treated with 20% NaCl and different pH (6 and 9). it was showed highly significant (*p*<0.001 or *p*<0.01) for antioxidant enzyme activity under abiotic stress . (Figure 2). A highly significant increase in SOD (*p*<0.001)(Figures 2A), CAT activity at 9 pH (Figure 2B). compared with the control and under salt and pH condition., in GSH highly (*p*<0.001) was observed under salt but modarate (*p*<0.01)(Figure 2C) at different pH (6,9).

### Plant Growth Promotion Activities in Maize Under *in vitro* Condition

Inoculation of different varieties of maize with PR19 *resulted* in a significant (*p* < 0.001) increase in percent seed germination, root and shoot length, and vigor index compared to control *in vitro* condition.

### Effect of PR19 on Morphological and Yield Parameters of Maize var. SHIATS MS-2 Under Pot Experiment

Based on *in vitro* studies, maize variety SHIATS-M S2 was selected for the pot experiment. This variety showed a high value of root (54 cm) and shoot (124 cm) elongation compared to other varieties. PR19 enhanced morphological, physiological, biochemical, and yield-related parameters in maize variety SHIATS-M S2.

**FIGURE 3:**
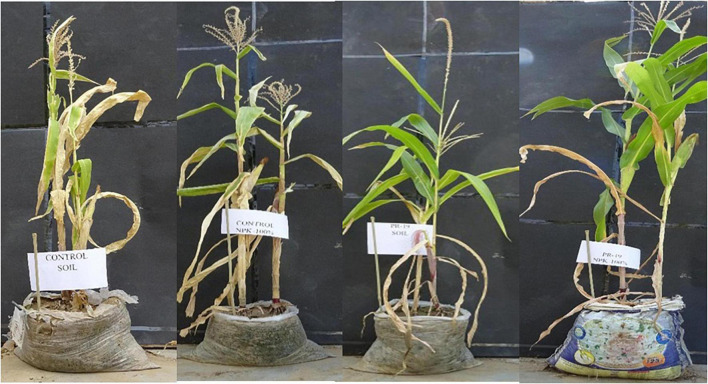
Effect of *PR19* on Maize Var. SHIATS MS-2 under pots/polybags condition. T0 = Control (no-fertilizer and no-bacteria inoculants), T1 = Std. NPK (120:60:40 kg/ha; 25%; no-bacteria inoculants only fertilizer), T2 = PR19 (only bacterial culture), T3 = PR19 (bacterial culture) and NPKs(120:60:40 kg/ha; 100%).

PR19 treatment significantly enhanced plant growth parameters in the maize variety SHIATS MS-2 compared to control plants with an increase of 11% in plant height, a 2-fold increase in flag leaf width, 48% in flag leaf length, 47% in cob length, 11% in cob weight, 4% in fresh weight, and 34% in dry weight of plant (Table 6). Similarly, physiological and biochemical parameters were enhanced in PR19 treated plants, as shown by 59% in total chlorophyll, 16% in relative water content, 59% in seed protein, and 32% in total carbohydrate (Table 7). Yield parameters showed a similar trend with an increase of 14% in grain yield per plant, 32% in 1000-grain weight, 11% in harvest index, and 5% in the number of grains per cob. The application of standard NPK (chemical) fertilizer resulted in a significant increase in all the above parameters compared with the control plants (Figure 3). PR19 alone showed a significant increase in all the parameters compared with NPK fertilizer except for an increase in the weight of cob and dry weight of the plant, which were not substantial. PR19 and NPK fertilizer significantly enhanced 12 out of 15 parameters compared with PR19 alone. Flag leaf width and total chlorophyll levels reduced, and the cob length did not change with PR19 and NPK fertilizer combination compared with PR19 alone.

**TABLE 6:** Effect of *Azotobacter nigricans* on growth parameters of different Maize verities.

Maize variety	Seed germination (% + SD)		Root length (cm + SD)		Shoot length (cm + SD)		Germination index (+SD)		Vigor index (+SD)	
					
	Control	PR19	Control	PR19	Control	PR19	Control	PR19	Control	PR19
Kanchan	52.3 + 2.5	53 + 2.6	4.3 + 0.25	8.2 + 0.3**	8.2 + 0.2	16.2 + 0.3***	3.5 + 0.1	5.4 + 0.2**	634 + 30.7	1297 + 89**
Azad Uttam	51.7 + 2.9	62 + 2.5**	4.1 + 0.1	10.2 + 0.3***	4.2 + 0.3	9.4 + 0.4***	3.6 + 0.2	5.7 + 0.1**	413.3 + 23.1	1248 + 95.4**
Saradmani	72.3 + 2.5	83 + 2.6**	4.16 + 0.2	10.4 + 0.4***	9.4 + 0.4	14.2 + 0.2**	4.3 + 0.2	6.8 + 0.1**	836 + 187	2043 + 108*
Navjyot	51.3 + 1.5	63 + 2**	24 + 1	35.7 + 2.1*	34.7 + 1.5	64 + 1**	4.3 + 0.2	6.53 + 0.1***	2977 + 88.5	6280 + 271**
SHIATS MS-2	72 + 2.64	63 + 2**	32.3 + 2.5	54 + 1**	85 + 1	124 + 1***	4.5 + 0.1	6.5 + 0.1*	4872 + 306	11217 + 482***

*p < 0.05, **p < 0.01, ***p < 0.001. AS: Ammonium sulfate.

**TABLE 7:** Effect of *Azotobacter nigricans* (KP966496; *PR19*) on morphological and yield parameters of maize var. SHIATS MS-2.

Parameters	Agronomic traits	T_0_	T_1_	T_2_	T_3_
Morphological	Plant height (cm)	190.3 ± 0.6a	200.3 ± 0.6b	209.6 ± 0.6***c	215.3 ± 0.6***d
	Flag leaf width (cm)	3 ± 0.05a	7.1 ± 0.1***b	9.0 ± 0.11***c	7.2 ± 0.17***b
	Flag leaf length (cm)	32.6 ± 1.2a	42.3 ± 0.6**b	48.3 ± 0.6***c	52.6 ± 2.1**d
	Length of cob (cm)	7.13 ± 0.2a	8.0 ± 0.05*a	10.3 ± 0.6*b	10.3 ± 0.6**b
	Weight of cob (g)	148.6 ± 0.6a	160.6 ± 1.2**b	164.3 ± 1.2***b	168.3 ± 0.6***c
	Fresh weight of plant (g)	360.6 ± 1.2a	370.3 ± 0.6**b	375.3 ± 0.6**c	385.3 ± 0.6**d
	Dry weight of plant (g)	204.6 ± 0.6a	270.6 ± 1.2***b	273.3 ± 1.5***b	280.6 ± 0.6***c
Physiological/Biochemical	Total Chlorophyll (mg/g)	20.3 ± 0.6a	28.3 ± 0.6b	32.3 ± 0.6***c	30.3 ± 0.6**d
	Relative water content (%)	67.6 ± 0.6a	75.3 ± 0.6**b	78.3 ± 0.6***c	85.6 ± 0.6d
	Protein (%) in seed	7.1 ± 0.1a	10.0 ± 0.11***b	11.3 ± 0.6**c	14.2 ± 0.17***d
	Total carbohydrate (%)	48.3 ± 0.6a	57.3 ± 0.6b	63.6 ± 1.15***c	66.3 ± 1.2**d
Yield	Grain yield per plant (g)	118.6 ± 0.6a	128.3 ± 0.6**b	134.6 ± 0.6c	140.6 ± 0.6d
	Average 1000 seed (g) weight	310.3 ± 0.6a	390.6 ± 1.2***b	410.3 ± 0.6***c	431.3 ± 1.15***d
	Harvest index (%)	131.6 ± 0.6a	142.3 ± 0.6***b	145.6 ± 1.2**c	150.6 ± 0.6***d
	Number of grains per cob	360.6 ± 0.6a	370.3 ± 0.6**b	377.3 ± 0.6***c	390.6 ± 0.6***d

*p < 0.05, **p < 0.01, ***p < 0.001 with reference to control.

*Values sharing different letters in the same row are significant with each other.*

## Discussion

Integrated nutrient management (INM) is a sensible application of fertilizers or manures from dissimilar sources to a field consecutively to preserve environmental sustainability. So the present study was planned to study the effects of *Azotobacter* on plant growth and production parameters alone and in combination with NPK fertilizers. Although the plant growth-promoting activity of *Azotobacter* has been well studied but there are only few reports for synergistic effects of the combination of chemical fertilizer and *Azotobacter* sp. (Kang et al., 2017; Prabhukarthikeyan et al., 2018). Inoculation of PR19 significantly improved maize yield, biochemical, and physiological parameters compared to control plants and plants with NPK fertilizer application. However, the dual application of PR19 and NPK chemical fertilizer gave better results (increased the grain yield) than a single use of PR19 and NPK alone. This indicated that PR19 could reduce the use of chemical fertilizers. It can be stated that the increase in growth parameters of maize is due to greater availability of nitrogen in full organic and integrated treatments. In full chemical treatments, most of the nitrogen would be leached from the soil profile. *A. nigricans* strain excretes extracellular phosphate solubilizing enzymes such as phytase (133UI in 48 h of fermentation) and phosphatase (170UI in 48 h of fermentation), which can solubilize the rock phosphate and make it available to plants. A significant increase in the concentration of phytase, phosphatase, and soluble phosphorus was also found after 48 h of fermentation along with a decrease in soluble phosphorus concentration. This decrease may be due to the utilization of phosphorous by fungus mycelia. Although fungi produce these enzymes to solubilize phosphate and phytic acid for their own growth. Consequently, a considerable amount of phosphorus becomes available to plants as well (Din et al., 2019).

Liang et al. (2015) demonstrated K concentrations in the liquid media were significantly higher than control, while the contents of soil mineral structure K were substantially lower in the *Azotobacter* treatments than in the non-*Azotobacter* treatment. Taking into account the soil being the sole K source, it is evident that *Azotobacters* could promote the dissolution of mineral K in the soil. The competence of *Azotobacter* species to solubilize K has been confirmed through several works (Archana et al., 2013; Diep and Hieu, 2013). Other works suggested that *Azotobacter* species can solubilize K and play a significant role in improving K absorption by plant (Singh et al., 2010; Baba et al., 2021).

1-aminocyclopropane1-carboxylate (ACC) deaminase is also a key trait produced by *Azotobacter* (Omer et al., 2016). ACC deaminase-producing organisms decrease plant ethylene levels which, when present in high concentrations, can lead to plant growth inhibition or even death (Honma and Shimomura, 1978; Glick et al., 2007). This enzyme is responsible for the cleavage of the plant ethylene precursor, ACC, into ammonia and -ketobutyrate by decreasing ACC levels in plants (Sagar et al., 2020b; Aasfar et al., 2021).

Saini et al. (2004), reported that a combination of 50 percent of chemical fertilizers and farm yard manure with inoculation of seeds by *Rhizobium* bacteria and phosphor solubilizing bacteria, increased grain yield and biomass of sorghum and chickpea. According to Akbari et al. (2009), an amalgamation of bio and chemical fertilizers augmented grain yield, plant height, biological yield, and harvest index of sunflower. Also, it has been shown that application of 50% N through chemical fertilizer + 25% through biocompost + 25% N through vermicompost significantly improved growth in terms of plant height, dry matter accumulation per plant, and LAI over the treatment of 100% N through chemical fertilizer. Moreover, the use of bio stimulators in conditions of environmental stress can shrink the effects of stress and boost soil water holding capacity, root growth and production (Santosh et al., 2013; Shirkhani and Nasrolahzadeh, 2016; Khan et al., 2017).

Recommendations of organic matter alone with synthetic fertilizers could be helpful for enhancing stagnant wheat grain yield Tahir et al. (2011). Similarly, Kemal and Abera (2015) reported application of a recommended dose of inorganic fertilizer along with vermicompost at 6 ton/ha to maize not only enhanced productivity of maize but also improved soil fertility in terms of higher available N, P, K, and organic carbon content over the control and recommended N, P and K. However, the gains were marginal, and the trade-off of not using chemical fertilizer is recommended. PR19 inoculation increased chlorophyll content related to higher photosynthesis, which in turn enhanced carbohydrate synthesis. Although the nitrogen content of maize kernels was not estimated in this study, several other studies showed that grain nitrogen levels are enhanced by PGPB, which results in higher protein content in grains (dos Santos et al., 2020; Yadav et al., 2020b).

The importance of *Azotobacter* as a microbial inoculant is convincingly established throughout various experiments and a large number of field trials. Ritika and Utpal (2014) showed in their review that the use of *Azotobacter* as an N-biofertilizer increased the growth and yield of various crops under field conditions with a percentage increase of up to 40% for Cauliflower and 15–20% for Maize compared to conventional fertilizers. These beneficial effects can be attributed to the biosynthesis of biologically active substances, the stimulation of rhizospheric microorganisms, the production of phytopathogen inhibitors, and improved nutrient availability of N, P, carbon, and sulfur, through BNF and mineralization of organic residues in soil (Lenart, 2012; Patel et al., 2017). Numerous studies described crop responses to *Azotobacter* inoculation under greenhouse and field conditions. Particularly, they are able to supply non-leguminous plants with a significant amount of N, in addition to synthesizing plant growth-promoting substances, which help increase the availability of additional nutrients (P, K, and Zn) for better plant nutrition.

## Conclusion

*Azotobacter nigricains* isolated in the present study produced multiple plant growth-promoting, biocontrol and salinity ameliorating traits. It enhanced plant growth through phosphate solubilization, siderophore, and ACC deaminase production. According to a field study, the isolate showed a positive impact on the agronomic traits of maize compared to chemical fertilizer. Under field conditions, the combined application of PR19 and NPK treatments showed similar enhancement in maize’s growth and yield parameters. Thus co inoculation of *A. nigricains* and NPK can serve as a dual-purpose or mixed fertilizer to enhance maize yield. The potential of this isolate could be further explored to enhance maize production following more studies in different agroclimatic zones.

## Data Availability Statement

The original contributions presented in this study are included in the article/supplementary material, further inquiries can be directed to the corresponding authors.

## Author Contributions

AS: conceptualization and methodology. AS, RS, PR, WR, and PP: writing the original draft. RS, PR, and PP: formal analysis. RS, PR, PP, SA and MA: writing – review and editing. SA: fund acquisition. PP: open funding. All authors have read and agreed to the published version of the manuscript.

## Conflict of Interest

The authors declare that the research was conducted in the absence of any commercial or financial relationships that could be construed as a potential conflict of interest.

## Publisher’s Note

All claims expressed in this article are solely those of the authors and do not necessarily represent those of their affiliated organizations, or those of the publisher, the editors and the reviewers. Any product that may be evaluated in this article, or claim that may be made by its manufacturer, is not guaranteed or endorsed by the publisher.
